# Early Life Exposure to Human Milk Oligosaccharides Reduces Allergic Response in a Murine Asthma Model

**DOI:** 10.1155/2023/9603576

**Published:** 2023-07-29

**Authors:** Tahereh Bozorgmehr, Rozlyn C. T. Boutin, Sarah E. Woodward, Katherine Donald, Jo May Chow, Rachael H. Buck, B. Brett Finlay

**Affiliations:** ^1^Michael Smith Laboratories, University of British Columbia, Vancouver, BC, Canada; ^2^Department of Microbiology and Immunology, University of British Columbia, Vancouver, BC, Canada; ^3^Nutrition Division, Abbott Laboratories, Columbus, OH, USA; ^4^Department of Biochemistry and Molecular Biology, University of British Columbia, Vancouver, BC, Canada

## Abstract

**Background:**

Studies suggest that early-life gut microbiota composition and intestinal short-chain fatty acids (SCFAs) are linked to future asthma susceptibility. Furthermore, infancy offers a critical time window to modulate the microbiota and associated metabolites through diet-microbe interactions to promote infant health. Human milk oligosaccharides (HMOs), nondigestible carbohydrates abundant in breast milk, are prebiotics selectively metabolized by gut microbiota that consequently modify microbiome composition and SCFA production.

**Methods:**

Using a house dust mite mouse model of allergy, we investigated the impacts of early oral treatment of pups with biologically relevant doses of 2′-fucosyllactose (2′-FL) and 6′-sialyllactose (6′-SL), two of the most abundant HMOs in human milk, in amelioration of allergic airway disease severity.

**Results:**

We found that administration of 2′-FL and 6′-SL during early life reduced lung histopathology scores, circulating IgE, cytokine levels, and inflammatory cell infiltration, all hallmark symptoms of allergic asthma. HMO supplementation also increased the relative abundance of intestinal *Bacteroidetes* and *Clostridia*, known SCFA producers within the gut. Indeed, we detected increased SCFA concentrations in both the intestine and blood of adult mice who received HMOs prior to weaning.

**Conclusion:**

We propose a model in which orally administered HMOs delivered during early life shift the microbiota toward increased production of SCFAs, which dampens the allergic immune responses behind allergy and asthma. Overall, these data suggest the potential for HMO supplementation to protect infants against asthma development later in life, with possible benefits against additional atopic diseases such as eczema and food allergies.

## 1. Introduction

The global prevalence of allergic asthma among children has substantially increased over recent years [1–3]. Although the etiology of asthma is not fully understood, this common chronic disorder of childhood is strongly associated with environmental, genetic, and nutritional factors which potentially alter both microbiota-derived metabolites and immune development during early life [1, 3–10]. Resident gut microbes, collectively termed the gut microbiota, are one of the major determinants influencing the gut metabolic profile and have many direct interactions with the host immune system [11, 12]. They are also one of the major producers of metabolites known as short-chain fatty acids (SCFAs), which have been shown to reduce allergic airway inflammation through the gut–lung axis [13]. Importantly, nutritional factors impact gut microbiota composition, affecting the crosstalk between microbiome-derived SCFAs and the host immune system and hinting at a possible mechanism of asthma development [1, 6, 14].

Human milk has a very unique composition and offers an optimal source of nutrients that enhances infant health, including protection against several types of diseases, such as asthma and allergy [5, 15–21]. Human breast milk is rich in a group of structurally complex unconjugated glycans known as human milk oligosaccharides (HMOs). Remarkably, HMOs represent the third largest solid component of breast milk, yet they are only minimally digested in the upper gastrointestinal tract before reaching the colon, where they serve as substrates for saccharolytic bacteria [11, 22–28]. HMOs that reach the colon intact can be fermented by certain species of gut bacteria to produce SCFAs [28], thereby impacting immune development and function. Following several studies which supported the potential protective effects of prebiotics in the development of allergy [11, 17, 29, 30], the biological function of HMOs is now of significant interest as a major component of human milk impacting human health.

To investigate the prebiotic potential of HMOs in amelioration of allergic airway disease severity, we assessed the impacts of 2′-fucosyllactose (2′-FL) and 6′-sialyllactose (6′-SL), the two predominant HMOs in breastmilk, on asthma development. Using a house dust mite (HDM) murine model of allergic asthma, we showed that HMO supplementation during early life leads to milder symptoms of allergic airway disease in adulthood. We further provided mechanistic insight by exploring microbiota composition in response to HMO treatment and identifying a shift toward increased production of immunomodulatory gut microbiota-derived SCFAs. Together, these data support the use of HMO supplementation in early life as a tool to improve infant health and reduce the risk of allergic disease.

## 2. Materials and Methods

### 2.1. Animal Housing and Ethics

C57BL/6J SPF pregnant (gestational days 11–15) mice were received from Jackson Laboratories (Bar Harbor, ME, USA) and maintained in the Modified Barrier Facility at the University of British Columbia. Dams received a breeder diet (Cat. #0007689, Lab Diet) upon arrival, and pups were maintained on a standard mouse chow (Cat. #0007688, Lab Diet) after weaning and throughout all experiments. Females and males were weaned at 3 weeks of age and randomly housed by sex at four per cage. Only female mice were used in this study [31]. All mice were maintained on a 12 hr light–dark cycle and provided with food and water *ad libitum*. All experiments were performed in accordance with the University of British Columbia Animal Care Committee guidelines and approved by the UBC Animal Care Committee (Animal Care Protocol A21-0286).

### 2.2. Neonatal Murine Exposure to HMOs

2′-FL and 6′-SL were provided by Abbott Laboratories (Columbus, OH, USA). Unless otherwise stated, 2′-FL and 6′-SL (500 mg/kg) were dissolved in phosphate buffer saline (PBS) and administered every other day by oral gavage to the pups from day 7 until day 21 of life. The control group of animals was gavaged with the vehicle. For the first experiment, 2′-FL or 6′-SL were provided via oral gavage from day 14 until day 20, followed by supplementing their drinking water with either 2′-FL or 6′-SL for a duration of 2 weeks after weaning. HMO-supplemented water was prepared fresh and changed every other day.

### 2.3. Asthma Allergy Model

This model is used to imitate human asthma by inducing allergic airway disease in mice. Asthma induction was initiated at 6 weeks of age, as previously described [32, 33]. To sensitize the mice, animals were anesthetized with 2.5% isoflurane before inoculation with 1 *μ*g of HDM protein (Greer Laboratories) in 40 *μ*L of PBS, which was applied intranasally using a P200 pipette on day 42 of the experiment. One week later, mice were anesthetized as before and intranasally challenged with 10 *μ*g of HDM protein in 40 *μ*L of PBS daily for 5 consecutive days (days 49–53). Three days after the final challenge (day 56), animals were humanely sacrificed by intraperitoneal injection of 500 mg/kg tribromoethanol (Avertin; Sigma). After the collection of blood via intracardiac puncture, bronchoalveolar lavage fluid (BALF), lung, and cecum tissues were collected. Fecal samples were collected on days 14, 21, 42, and 56 of the experiment for 16S ribosomal RNA (rRNA) analysis of gut microbial composition and SCFA measurement.

### 2.4. Cellular Infiltration of the Airways

The cellular compartment of the airway lumen was assessed by BALF collection. Lungs were washed through the trachea by injection of 1 mL PBS containing a complete ethylenediaminetetraacetic acid (EDTA)-free protease inhibitor cocktail (Roche Diagnostics) as recommended by the manufacturer. Total cell numbers were counted with a hemocytometer. Differential cell counts were performed by preparing BALF cytospins (700 rpm for 5 min, with low acceleration, Thermo Shandon Cytospin 3 Centrifuge-Marshall Scientific), which were stained with Diff-Quik solution kit (RAL Diagnostics, 3 × 100 mL). Percentages of eosinophils and neutrophils were determined by counting 50 cells per sample.

### 2.5. Lung Histology

Lung segments were collected and immediately fixed in 10% formalin for 24–48 hr. Tissues were longitudinally sliced into 5 mm sections after washing with 70% ethanol. Sections were embedded in paraffin, followed by staining with hematoxylin and eosin or periodic acid-Schiff (PAS) by Wax-It Histology Services (Vancouver, Canada). Histological analysis of lung tissues was assessed blindly as previously described [34, 35] with minor modifications. Specifically, for lung histopathology scoring, each section was scored from 1 to 5 (1 = no signs of disease; 5 = severe disease) using a 4x objective for each of the following parameters: (1) peribronchial infiltration, (2) perivascular infiltration, (3) parenchymal infiltration, and (4) epithelium damage for a maximum score of 20.

Mucus production was assessed by quantification of the goblet cell hyperplasia in the airway epithelium using a four-point grading system: (1) no goblet cells or less than 25%, (2) 25%–50%, (3) 50%–75%, and (4) 75% and more. Scoring of the goblet cells was performed in at least five different fields for each lung section. Mean scores were obtained from 24 animals per group.

### 2.6. Quantitation of SCFAs

Measurement of SCFA concentration was performed by the UVic-Genome BC Proteomics Centre as previously described [36] with minor modifications. Briefly, a standard substance solution of SCFAs was serially diluted to have 10 working standard solutions. Sixty percent acetonitrile was added to each fecal and cecal sample, followed by homogenization and sonication. Serum samples were mixed with acetonitrile and sonicated in an ice-water bath. All samples were centrifuged, and the supernatants were used to run liquid chromatography-multiple reaction monitoring/mass spectrometry on an Agilent 1290 ultra-high-performance liquid chromatography system coupled to an Agilent 6495B QQQ mass spectrometer.

### 2.7. Fecal DNA Extraction and 16S Analysis

DNA isolation, polymerase chain reaction amplification, sequencing, and sequence processing were performed by Microbiome Insights (Vancouver, Canada). Briefly, fecal DNA was extracted by using MoBio's instructions on a KingFisher robot. Dual-barcoded primers, which targeting the V4 variable region (515 F 5′-GTGCCAGCMGCCGCGGTAA-3′, and 806R 5′-GGACTACHVGGGTWTCTAAT-3′) were used to amplify bacterial 16S rRNA, as described by Kozich et al. [37]. Amplicon sequencing was performed on a MiSeq platform (Illumina) using a 300 bp paired-end kit (v.3).

Sequences were taxonomically classified using Silva (v. 138) and clustered into 97%-similarity operational taxonomic units with the mothur software package (v. 1.44.1) developed by Kozich et al. [37]. The procedure was followed according to https://www.mothur.org/wiki/MiSeq_SOP website, which was assessed in Nov 2020.

### 2.8. Cytokine and IgE Determination

Cytokine concentrations were measured in lung tissues using commercially available enzyme-linked immunosorbent assay (ELISA) kits (ThermoFisher Scientific). Lung tissues were collected from animals and immediately stored in PBS with a complete EDTA-free protease inhibitor cocktail (Roche Diagnostics) at the final concentration recommended by the manufacturer. All tissues were weighed before being homogenized with a MixerMill 301 bead miller (Reutsch) for 2 min at room temperature. The homogenized tissues were centrifuged at 15,000 rpm (21,660 × *g*) for 10 min at 4°C, and the resulting supernatants were stored at −70°C until use. IL-4, IL-5, IL-10, and IL-17 cytokine levels in the lung homogenates were measured using ELISA kits (ThermoFisher Scientific) according to the manufacturer's recommendations. Absorbance at 450 nm was measured using a Synergy H1 plate reader (Biotek).

Cytokine levels (IL-4 and IL-6) in the BALF supernatants were screened using a Cytometric Bead Array Mouse Inflammation Kit (BD Biosciences) according to the manufacturer's recommendations.

In order to quantify serum IgE levels, mice were sacrificed as described above, and blood was collected thereafter via cardiac puncture. Serum was separated from clotted blood by centrifugation at 1,100 rpm for 10 min. Total serum IgE and HDM-specific IgE were measured by ELISA (ThermoFisher Scientific and MyBioSource) according to the manufacturer's instructions. Absorbance at 450 nm was measured using a Synergy H1 plate reader (Biotek).

### 2.9. Statistical Analysis

Statistical analysis was performed using GraphPad Prism9 or RStudio. Group comparisons were determined by Mann–Whitney nonparametric tests, and error bars represented the mean ± SEM of the pooled data unless otherwise indicated. For the microbiome data, alpha diversity was analyzed using a Wilcoxon Rank-Sum test. The differences in beta diversity of the groups were assessed by using a permutational analysis of variance (PERMANOVA) by the adonis2 function in the package vegan 2.6-2 (https://cran.r-project.org/web/packages/vegan/index.html). Statistical significance is presented by either *p*-values or  ^*∗*^*p* < 0.05,  ^*∗∗*^*p* < 0.01, and  ^*∗∗∗*^*p* < 0.001.

## 3. Results

### 3.1. 2′-FL and 6′-SL Influence Susceptibility to Allergic Airway Inflammation

Previous studies have illustrated that 2′-FL and 6′-SL are capable of reducing food allergy symptoms in mice [11, 12]. However, no data have yet been reported regarding the impact of these two HMOs on symptoms of airway inflammation, such as those observed in murine models of asthma.

**Figure 1:**
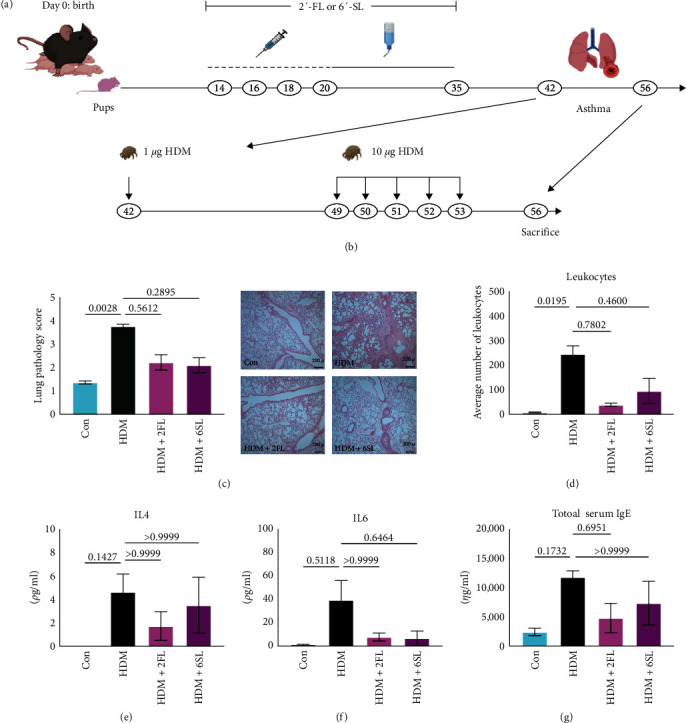
Mice exposed to 2′-FL or 6′-SL at early-life demonstrate dampen inflammatory responses during allergic airway disease later in life. (a) Mice experimental setup illustrating the dosing regimen of HMOs up to 5 weeks of age and (b) house dust mite (HDM) model of allergic airway disease. (c) Lung histopathology scores (left; 1–5) and representative images (right; 4x objective). (d) Quantification of the total number of Leukocytes in the bronchoalveolar lavage fluid (BALF). (e, f) The concentration of IL-4 and IL-6 in the BALF. (g) ELISA detection of serum IgE. Con, control group; HDM, asthmatic group. Each group includes four mice. Statistical significance determined by Mann Whitney nonparametric test and bar graph indicates the mean with SEM.

In order to examine whether HMOs are effective in reducing allergic inflammation observed in asthma, we designed a study to expose mouse pups to HMOs, followed by the induction of asthma-like symptoms in adulthood. To do this, 14-day-old female pups were exposed to 2′-FL, 6′-SL, or PBS control every other day until the last day of the lactation (day 20). After weaning, HMOs were administered via drinking water for two additional weeks (Figure 1(a)). At 6 weeks of age, which represents sexual maturity, asthma was induced via HDM sensitization and challenges followed by monitoring of ensuing allergic airway inflammation (Figure 1(b)).

First, we assessed the tissue pathology of mice that received HMO and those that received the PBS control to compare differences in the overall severity of allergic inflammation. Histological analysis of lung tissues indicated a trend toward lower inflammatory pathology scores in the airways of the mice which received HMOs (Figure 1(c)). A similar trend was observed upon quantification of inflammatory immune cell infiltration in the lungs represented by a total number of leukocytes (Figure 1(d)). Consistent with the decreased leukocyte infiltration, concentrations of IL-4 and IL-6 were also reduced in the fluid collected from the lung of HMO-treated mice (BALF; Figures 1(e) and 1(f)). Furthermore, total serum IgE levels decreased in the HMO-treated animals compared to the PBS-treated control mice (Figure 1(g)), indicating the possible effects of HMO supplementation on a global inflammatory state. Although we did not obtain significant differences between treated and nontreated groups due to the small sample size (four mice per group), these results supported the potential for 2′-FL and 6′-SL to influence the overall severity of the inflammatory response in allergic asthma.

### 3.2. Early Life Exposure to 2′-FL and 6′-SL in Combination Strongly Attenuates Allergic Response Later in Life

**Figure 2:**
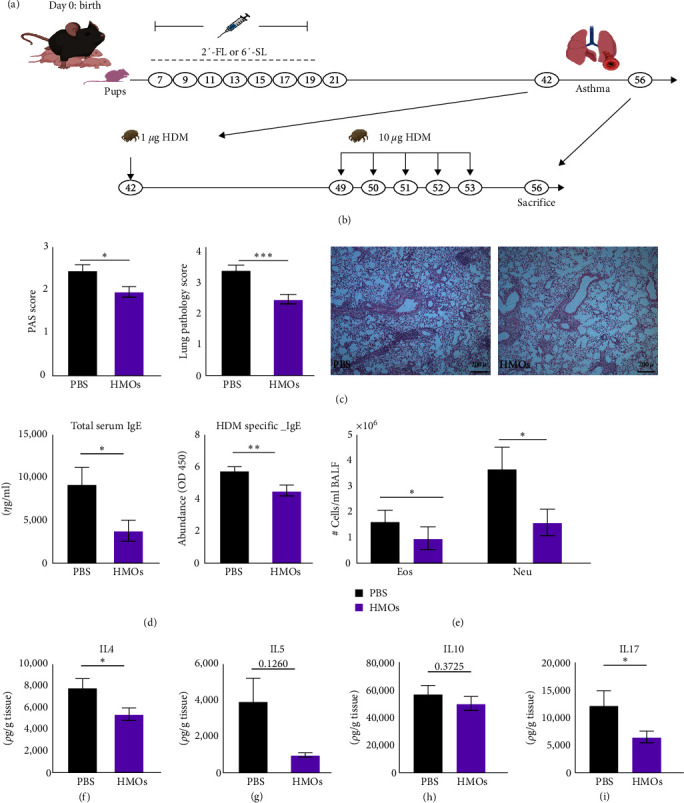
Preweaning exposure to HMOs reduced HDM-induced lung inflammation later in life. (a) Mice experimental setup illustrating the dosing regimen of HMOs up to 3 weeks of age and (b) house dust mite (HDM) model of allergic airway disease. (c) PAS score of lung tissues (right; 1–4; PBS *n* = 24, HMOs *n* = 24), lung histopathology scores (middle; 1–5; PBS *n* = 24, HMOs *n* = 24), and representative images (right; 4x objective). (d) ELISA detection of total serum and HDM-specific IgE (PBS *n* = 18, HMOs *n* = 17). (e) Quantification of the eosinophils (Eos) and neutrophils (Neu) in the bronchoalveolar lavage fluid (BALF; PBS *n* = 13, HMOs *n* = 17). (f–i) Cytokine levels in the lung tissue (PBS *n* = 18–24, HMOs *n* = 18–24). Data are pooled from either two or three independent experiments each showing the same trends. Statistical significance determined by Mann Whitney nonparametric test, bar graph indicates the mean with SEM ( ^*∗*^*P* < 0.05,  ^*∗∗*^ < 0.01,  ^*∗∗∗*^ < 0.001).

Considering the promising results of this initial study, we next sought to adapt the experimental setup to provide HMO exposure to younger mice as being more reflective of mouse–human ages and furthermore to better reflect the fact that breastfed human infants would not receive HMOs after being weaned. Therefore, follow-up experiments were done using a modified procedure such that pups were only exposed to HMOs from days 7 to 21 following birth. Furthermore, since both 2′-FL and 6′-SL showed positive effects on dampening asthma symptoms in the pilot study, we decided to use a combination of 2′-FL and 6′-SL to maximize the impact of the two individual HMOs on asthma symptoms. Pups were exposed every other day to either the combination of 2′-FL and 6′-SL or to PBS control via oral gavage starting at 7 days of age up until the last day of lactation (day 21; Figure 2(a)). The PBS and HMO-treated mice were again intranasally sensitized and challenged with HDM extract starting at 6 weeks of age (Figure 2(b)). As anticipated, we found that early life exposure to 2′-FL and 6′-SL resulted in a significant reduction in the development of allergic airway inflammation during adulthood as evidenced by lung goblet cells and histopathology scores (*p* < 0.05 and *p* < 0.001; Figure 2(c)), total and HDM specific circulating IgE (*p* < 0.05 and *p* < 0.01; Figure 2(d)), lung-infiltrating eosinophils and neutrophils (*p* < 0.05; Figure 2(e)), and cytokine levels (*p* < 0.05; Figure 2(f)–2(i)) in lung tissues. Together, these data demonstrate the potent ability of HMO supplementation to ameliorate allergic airway inflammation.

### 3.3. Early Life Exposure to 2′-FL and 6′-SL Has Lasting Effects on Gut Microbial Composition

Recent evidence indicates that breastfeeding during the first few months of a newborn's life shapes their intestinal microbiota ecology [5, 6], which further influences immune cell homeostasis and possibly modulates susceptibility to allergic inflammation later in life [14, 38]. In order to determine whether early life exposure to HMOs has a long-term impact on gut microbiota composition, mouse feces were collected starting seven days after 2′-FL and 6′-SL exposure (day 14). Fecal pellet collection was repeated on the day of weaning (day 21), before asthma induction (day 42), and on the last day of the experiment (day 56). The gut microbiota composition of the animals was then assessed over time by 16S sequencing of fecal samples.

**Figure 3:**
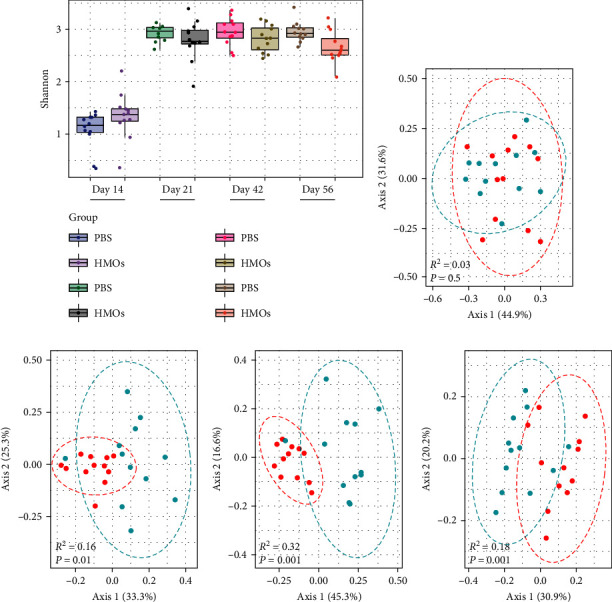
Mice treated with HMOs in the early stage of life exhibit alterations to their gut bacterial populations. (a) Alpha diversity (Shannon index) derived from 16S rRNA gene amplicon sequencing of fecal samples collected at 14, 21, 42, and 56 days of age from mice treated with PBS or HMOs (PBS *n* = 12; HMOs *n* = 12). Wilcoxon test determined significant differences in the Shannon diversity index. Dots represent individual mice. (b–e) Principal coordinate analysis (PCoA) plot based on Bray–Curtis dissimilarity distances from 16S rRNA gene sequencing data from fecal samples collected at 14, 21, 42, and 56 days of age (PBS *n* = 12; HMOs *n* = 12). *p*-Values determined by PERMANOVA.

We first evaluated the alpha diversity within samples, as measured with the Shannon Index, which we found to be similar between the PBS- and HMO-treated mice on days 14, 21, 42, and 56 of the experiment (Figure 3(a)), indicating that early life HMO exposure has little impact on the richness and diversity of gut bacterial species. With regard to beta diversity between samples, we found that bacterial populations in the gut of the HMO- and PBS-exposed mice were not significantly different at 14 days of age (*p* > 0.05; Figure 3(b)) by principal coordinate analysis according to Bray–Curtis dissimilarity. However, the composition between the two treatment conditions started to diverge at day 21 of life (*p* = 0.01; Figure 3(c)), and these differences persisted beyond the duration of HMO exposure until the last day of the experiment (day 42, *p* = 0.001; day 56, *p* = 0.001; Figures 3(d) and 3(e)). These data suggest that differences in the gut bacterial populations at the time of asthma induction might be linked to the observed reduction in the severity of airway inflammation in the group of animals who received HMOs earlier in life.

**Figure 4:**
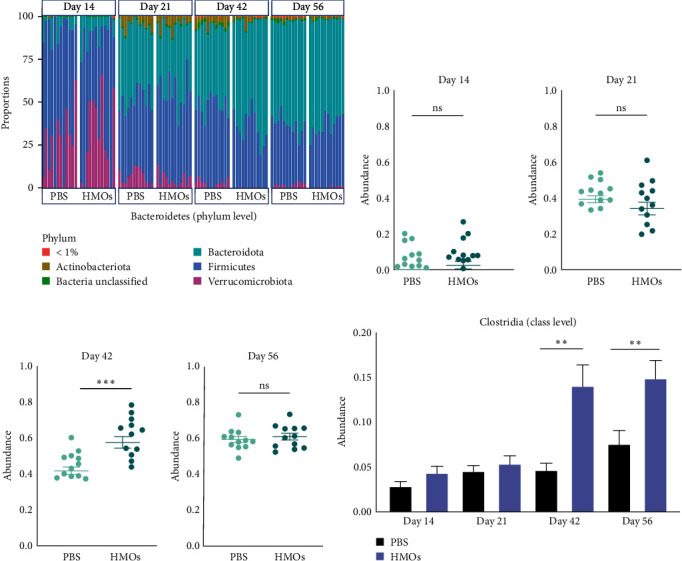
Preweaning exposure to HMOs altered the intestinal microbiota composition. (a) Relative abundance of operational taxonomic units (OTUs) of fecal samples collected on days 14, 21, 42, and 56 of experiment. Mice were treated with either HMOs or PBS, and colors are corresponded to phyla. (b–f) Relative abundance of Bacteroidetes (Phylum level) and Clostridia (class level) on day 14, 21, 42, and 56 of experiment from the mice treated with either PBS or HMOs at the early stage of life (PBS *n* = 12; HMOs *n* = 12). Statistical significance for (b–f) determined by Mann–Whitney nonparametric test, bar graph indicates the mean with SEM ( ^*∗∗*^*P* < 0.01,  ^*∗∗∗*^ < 0.001).

In order to determine which bacteria were altered in relative abundance as a result of HMO treatment, we performed further analysis of the 16S rRNA genes of fecal microbiota at various taxonomic levels. This analysis revealed alterations in the composition of the gut microbiota over time depending on the treatment condition. One week after receiving HMOs (day 14 of life), no clear differences were detected between groups at the phylum level, reflecting the beta diversity data (Figure 4(a)). However, while the proportion of *Bacteroidetes* bloomed in both groups by day 21 (Figure 4(a)), the *Bacteroidetes* expanded more rapidly in the HMO-treated group relative to the PBS-treated group (*p* < 0.001; Figures 4(a) and 4(d)). Specifically, the abundance of the *Bacteroidetes* surged to an average relative abundance of 60% in HMO-treated mice by day 42, while the PBS group reached the same level only by day 56 (Figure 4(b)–4(e)).

Taxonomic analysis at the class level also indicated a remarkable difference in the relative abundance of *Clostridia* between experimental groups. We found that 3 weeks after the last HMO treatment (day 42), the relative abundance of *Clostridia* (class level) in the HMO-treated animals substantially increased in comparison with the mice that received PBS only, and this expansion persisted up until the last day of the experiment (*p* < 0.01; Figure 4(f)). *Clostridia* have been previously reported to produce SCFAs and have systemic anti-inflammatory effects [39], which suggests that the milder symptoms of asthma in the group receiving HMOs might be related to the marked shifts in the population of *Clostridia* in response to 2′-FL and 6′-SL supplementation.

### 3.4. Early Life Administration of HMOs Impacts SCFA Concentrations during Adulthood

Accumulating evidence suggests HMOs influence not only the gut microbiota population but also their fermentation processes [28, 40]. Furthermore, studies indicate that SCFAs, which are products of microbial fermentation of carbohydrates, including HMOs, are linked to the development of asthma and other chronic inflammatory disorders [1, 8]. Given the increased relative abundance of both *Bacteroidetes* and *Clostridia* in the fecal microbiota of HMO-treated animals at the time of asthma induction, both of which are rich in SCFA-producing taxa [41, 42], we hypothesized that commensal bacteria-derived SCFA production in early life might be involved in beneficial immunomodulatory and asthma-protective effects observed later on.

**Figure 5:**
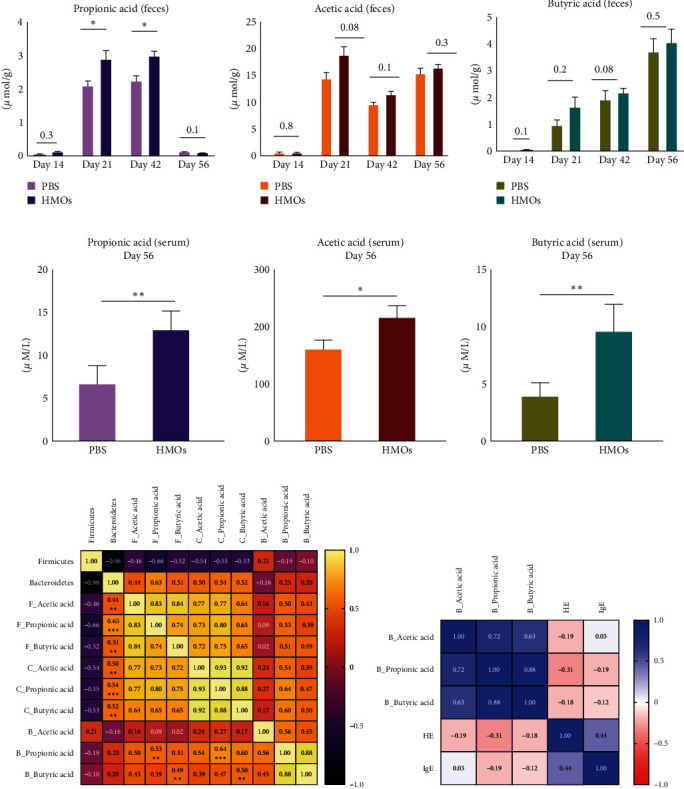
Treatment with HMOs altered microbial metabolites in the gut and serum. (a–f) The concentration of the three most abundance short-chain fatty acids (propionic, acetic, and butyric) in (a–c) feces (PBS *n* = 12; HMOs *n* = 12) and (d–f) serum (PBS *n* = 18; HMOs *n* = 18) from the asthmatic (PBS) and asthmatic HMO-treated mice. (g) Concentrations of the SCFAs from day 56 in the cecum and feces of the HMOs-treated mice were positively correlated with the abundance of Bacteroidetes in the gut (*n* = 35). (h) Concentrations of the propionic acid in the blood were negatively correlated with the histopathology score (HE) of the lung, and the serum IgE and HE show a positive correlation on the last day of experiment (*n* = 24; F, feces; C, cecum; B, blood). Spearman correlation between samples is displayed in the heatmaps. Statistical significance determined by Mann–Whitney nonparametric test, bar graph indicates the mean with SEM ( ^*∗*^*p* < 0.05,  ^*∗∗*^ < 0.01).

To assess whether exposure to HMOs at an early age impacts levels of SCFAs later in life, we measured the concentration of the three most abundant SCFAs in the gut, acetic acid, propionic acid, and butyric acid, in the feces of the animals on days 14, 21, 42, and 56 of the experiment. In addition, concentrations of the SCFAs in the cecum and serum of the animals were measured on the last day of the experiment (day 56). We observed that the levels of propionic acid in the feces of HMO-treated mice were significantly higher compared to levels in nontreated animals on days 21 and 42 (day 21, *p* = 0.03; day 42, *p* = 0.01; Figure 5(a)) but not on day 56, suggesting that increased levels of propionic acid before asthma induction might be associated with prevention of airway inflammation during adulthood. Although we observed a strong trend towards higher acetic acid (Figure 5(b)) and butyric acid (Figure 5(c)) concentrations in the feces of HMO-treated mice on days 21, 42, and 56, the results were not significantly different between groups. However, we found that concentrations of all three SCFAs were significantly increased in the serum of HMO-treated mice on the last day of the experiment (day 56) (Figure 5(d)–5(f)).

In order to link intestinal SCFA levels with the gut microbiota, we compared phylum-level bacterial abundance with the concentration of SCFAs measured across samples. Our results indicated that the population of the *Bacteroidetes* was positively correlated with the concentration of SCFAs in the cecum and feces of the animals on day 56 (Figure 5(g); Table 1). In addition, we were able to demonstrate a positive association between levels of propionic and butyric acid in the feces and cecum with concentrations of the same SCFAs in the blood on day 56 of the experiment (Figure 5(g); Table 2). We also detected a nonsignificant negative correlation between the histopathology scores of the lung tissues and blood propionic acid, as well as a positive correlation between the histopathology score of the lungs and the serum IgE levels at sacrifice (Figure 5(h); Table 3). Together, these data suggest that HMO supplementation prior to weaning promotes *Bacteroidetes* in the gut and thus increases circulating levels of the propionic acid, leading to a reduction in allergic inflammation later in life.

## 4. Discussion

To our knowledge, there are only a few human studies to date that have investigated the potential impacts of HMOs on allergic airway inflammation during childhood [5, 43]. Thus far, findings between studies have been inconsistent due to disparities described in their study settings, methodological limitations, and variations in the composition of the human breastmilk [5]. To overcome some of these limitations associated with studies in humans, we used an animal model of allergic airway inflammation to mechanistically link the administration of 2′-FL and 6′-SL during the lactation stage of life with the attenuation of allergic airway inflammation in adulthood. While several studies have demonstrated that nondigestible oligosaccharides ameliorate allergic reactions in murine models of food and asthma allergy [11, 12, 44], ours is the first to investigate the effects of HMO supplementation during the early life window, mimicking breast milk exposure, and demonstrating benefits to health long after the cessation of treatment.

Over recent years, growing evidence has indicated an association between gut microbiota composition and airway inflammation among breastfed infants [1, 45]. Furthermore, it has been reported that dietary fiber consumption suppresses asthma symptoms in multiple murine models of allergy, potentially through reshaping the gut microbiota population with subsequent changes in immune regulations and anti-inflammatory responses [14, 38, 46]. Interestingly, previous studies report that an increased proportion of *Bacteroidetes* in the gut leads to decreased susceptibility to airway inflammation [46]. The 16S analysis of the gut microbiota from our study demonstrates that 2′-FL and 6′-SL substantially promote the expansion of *Bacteroidetes* and *Clostridia* species. Our findings are consistent with observations by others that *Bacteroidetes* can metabolize HMOs and thereby directly influence the composition of the infant gut microbiota [28, 47–49].

In contrast to *Bacteroides*, *Clostridia* strains have been reported as non-HMO consumers [47, 48], suggesting that the outgrowth of *Clostridia* following HMO supplementation might result from the presence of a “cross-feeding” effect. In fact, researchers have previously shown that the intermediate degradation products of the HMO-utilizing *Bacteroidetes*, such as lactate and acetate, can be consumed by *Clostridia* strains to provide them with a competitive advantage within the gut environment [50]. As such, we propose that the early life consumption of 2′-FL and 6′-SL by select members of the gut microbiota leads to the production of intermediate breakdown products, which serve as substrates to support the expansion of *Clostridia* strains later in life.

*Clostridia* have long been associated with human health, particularly through the ability of some *Clostridia* species to provide protective “colonization resistance” against pathogen invasion, and this is especially important during the early stages of life [51]. Furthermore, the proliferation of *Clostridia* in the colon has been linked to reduced symptoms of colitis and allergy, possibly through the regulation of immune homeostasis [52, 53]. These reports support our findings that animals with a higher intestinal abundance of *Clostridia* exhibit reduced airway inflammation. Nevertheless, further research seems necessary to shed light on the possible associations between HMOs, *Clostridia* species, and asthma.

Emerging evidence highlights a pivotal role of gut microbiome-derived SCFAs in several diseases beyond the gastrointestinal tract, including diabetes, rheumatoid arthritis, allergy, and asthma [1, 6, 38, 54, 55]. SCFAs are well-known for their immunomodulatory properties, and increasing attention has been given to their potential roles in protecting human infants against the development of allergy [17, 56]. Our findings indicate that early life exposure to HMOs leads to a long-lasting effect on SCFA concentrations both within the gut and the systemic circulation, associated with shifts towards an SCFA-producing microbiota, which might influence immune system development and ultimately reduce symptoms of asthma later in life. Indeed, we identified a positive correlation between the proportion of *Bacteroidetes* and concentrations of acetic acid, propionic acid, and butyric acid in the cecum and feces of the animals at the experimental end. Our results also indicate that circulating levels of the three major SCFAs substantially increased in the HMO-treated animals, suggesting potential systemic effects on the host.

In summary, our findings highlight the potential for HMO supplementation during early life to reduce the symptoms and inflammatory response associated with allergic asthma, potentially acting through modifications to the gut microbiota composition, which result in the increased production of immunomodulatory gut microbiota-derived SCFAs. Dietary prebiotic supplementation has been proposed as a more reliable way to shift the human microbiome toward a health-associated state, as opposed to direct administration of probiotic strains, which often require repeated administration and demonstrate transient colonization at best [57]. Several animal studies indicate that a high-fiber diet modifies gut microbiota composition, which similarly increases SCFA concentrations and also results in decreased symptoms of airway inflammation [14, 38]. The data presented in this study further supports the use of HMO supplementation in early life as a potential strategy to improve infant health. Further studies are needed to investigate the mechanisms of action underlying the observed preventative effects of HMOs on the development of asthma.

## Figures and Tables

**Table 1:** Correlations between fecal and cecum SCFA concentrations and abundance of Bacteroidetes on day 56. Associations determined using Spearman correlation analysis (*n* = 35).

	Bacteroidetes	Spearman *r*	*p*-Value	95% CI of *r*s
Acetic acid	Feces	0.44	**0.0075**	0.1195 to 0.6824
	Cecum	0.5	**0.0022**	0.1906 to 0.7194

Propionic acid	Feces	0.63	**0.00004**	0.3732 to 0.8025
	Cecum	0.54	**0.0008**	0.2396 to 0.7433

Butyric acid	Feces	0.51	**0.0018**	0.2014 to 0.7248
	Cecum	0.52	**0.0014**	0.2142 to 0.7311

Bold values signify the significant *p*-Value.

**Table 2:** Correlations between fecal and cecum SCFA concentrations and levels of the same SCFAs in the blood on day 56. Associations determined using Spearman correlation analysis (*n* = 35).

	Blood	Spearman *r*	*p*-Value	95% CI of *r*s
Acetic acid	Feces	0.16	0.36	−0.1949 to 0.4746
	Cecum	0.23	0.17	−0.1152 to 0.5354

Propionic acid	Feces	0.53	**0.001**	0.2318 to 0.7396
	Cecum	0.64	**0.00003**	0.3857 to 0.8076

Butyric acid	Feces	0.49	**0.003**	0.1741 to 0.7111
	Cecum	0.5	**0.002**	0.1924 to 0.7203

Bold values signify the significant *p*-Value.

**Table 3:** Correlations between histopathology scores of the lung and levels of the propionic acid and IgE in the blood. Associations determined using Spearman correlation analysis (*n* = 24).

	HE	Spearman *r*	*p*-Value	95% CI of *r*s
Propionic acid		−0.31	0.14	−0.6434 to 0.1162
IgE		0.44	**0.04**	0.01788 to 0.7260

Bold values signify the significant *p*-Value.

## Data Availability

The data that support the findings of this study are available from the corresponding author upon reasonable request.
